# Improving delirium knowledge and recognition confidence in nursing homes through an e-learning program: a pre–post study

**DOI:** 10.1186/s12909-026-09297-2

**Published:** 2026-04-25

**Authors:** Vincent Molitor, Johanna Christina Seiters, Jonas Hoch, Petra Thürmann, Bernhard Holle, Horst Christian Vollmar, Rebecca Palm

**Affiliations:** 1 Division of Nursing Science, Department for Health Services Research, School of Medicine and Health Sciences, Carl von Ossietzky Universität Oldenburg, Ammerländer Heerstraße 140, Oldenburg, 26129 Germany; 2 Department of Primary care and Health Services Research, Nursing Science and Interprofessional Care, Medical Faculty Heidelberg, University Heidelberg, Im Neuenheimer Feld 130.3, Heidelberg, 69120 Germany; 3 Department of Medicine, Chair of Clinical Pharmacology, Faculty of Health, Witten/Herdecke University, Alfred-Herrhausen-St. 45, Witten, 58455 Germany; 4 German Center for Neurodegenerative Diseases (DZNE), Witten, Stockumer St. 12, Witten, 58453 Germany; 5 Institute of General Practice and Family Medicine (AM RUB), Faculty of Medicine, Ruhr University Bochum, Universitätsstr. 150, Bochum, 44801 Germany

**Keywords:** Computer-assisted instruction, Delirium, Education, distance, Geriatric nursing, Long-term care, Nurses, Nursing homes

## Abstract

**Background:**

Delirium is a neuropsychiatric syndrome associated with serious complications. Residents of nursing homes are particularly vulnerable to developing delirium. Nurses play a key role in the prevention, detection, and management of delirium but often report a lack of specific knowledge and understanding. The aim of this study was to develop and evaluate a tailored e-learning programme on delirium for nurses working in German nursing homes.

**Methods:**

This pilot study employed a single-group pre-post design. Between January and March 2025, nurses from German nursing homes completed a delirium-specific e-learning program consisting of five modules on risk factors, causes, prevention, symptoms, diagnosis, and treatment. Participants completed a delirium knowledge questionnaire and rated their confidence in recognizing delirium before and after training. Evaluation questions assessed usability and relevance.

**Results:**

A total of 80 nurses completed the e-learning program as well as the pre- and posttests. Delirium-specific knowledge improved from a median of 32 to 40 correct answers (interquartile range [IQR] = 9.25 vs. 7.5; *p* < 0.001). Subjective confidence in recognizing delirium increased from 5 to 7 (IQR = 2.25 vs. 1; *p* < 0.001) on a 10-point scale. Both improvements showed large effect sizes (knowledge: *r* = 0.86; subjective confidence: *r* = 0.92). Most nurses rated the training as highly relevant and usable.

**Conclusions:**

Residents of nursing homes are at increased risk of delirium, while nursing staff report a clear need for delirium-specific expertise. Findings from this pilot study suggest that the tailored e-learning program may enhance nurses’ knowledge and confidence in recognizing delirium. The program represents a promising foundation for structured delirium training in nursing homes and warrants further evaluation in larger and long-term studies.

**Supplementary Information:**

The online version contains supplementary material available at 10.1186/s12909-026-09297-2.

## Background

Delirium is an acute neuropsychiatric syndrome that constitutes a medical emergency. In the Diagnostic and Statistical Manual of Mental Disorders, Fifth Edition, Text-Revision (DSM-V-TR), delirium is classified as a neurocognitive disorder that is diagnosed when attention and consciousness are impaired, along with cognitive performance. Furthermore, delirium develops rapidly, fluctuates in severity throughout the day, and is a direct physiological response to a medical condition, substance intoxication, substance withdrawal, or other cause that cannot be explained by a preexisting neurocognitive disorder such as dementia [1]. Delirium is associated with numerous serious complications, such as an increased risk of hospitalization and mortality [2, 3]. In addition, delirium can promote the onset of dementia or exacerbate existing dementia [4]. For affected individuals and their families, delirium is an emotionally stressful experience [5]. Apart from the individual effects, delirium also has health related economic consequences resulting from hospitalizations and expected follow-up costs [6].

Delirium research has focused primarily on hospitals, where proven delirium prevention and management programs already exist. Nursing homes, in contrast, have received less attention, and the existing delirium prevention programs in hospitals cannot be easily transferred to this setting [7]. In general, there is little evidence of effective interventions to prevent and treat delirium in nursing homes [8].

This is a critical issue, as nursing home residents are particularly vulnerable to developing delirium because of their advanced age, preexisting cognitive impairment, and the effects of taking multiple medications [9–12]. The international prevalence estimates for delirium in nursing homes vary between 1.4% and 70.3% [13]. The first prevalence study on delirium in nursing homes in Germany is currently being conducted [14]. Given the high vulnerability of nursing home residents and the substantial variability in reported prevalence rates, increased awareness and structured knowledge regarding delirium in this setting are particularly important. However, the phenomenon of delirium in nursing homes appears to be structurally neglected, and the knowledge of delirium among nursing staff in this high-risk environment is limited [15].

Nursing staff in nursing homes play a key role in delirium care practices, as they must be able to recognize sudden changes in residents’ behavior, then notify responsible general practitioners and initiate initial nonpharmacological measures for delirium treatment. However, nursing staff rarely use the term “delirium” and describe making the distinction between delirium, dementia, and depression as difficult [16]. In addition, there are knowledge gaps in practical nursing care in the areas of prevention, diagnosis, treatment, and management of delirium, thus, structured nursing practices are lacking [17], even when guidelines exist [18]. The limited knowledge and lack of attention to delirium among nurses in nursing homes mean that many episodes of delirium are not recognized or are interpreted as signs of a physiological ageing process or dementia, obscuring the urgency of the delirium emergency. Therefore, there is a need for delirium-specific training for nurses in nursing homes.

To develop an education program on delirium in nursing homes that is as accessible as possible and allows flexibility and independent learning, we developed an asynchronous e-learning program. The umbrella term e-learning refers to the use of technologies to support learning activities [19]. Asynchronous learning is particularly suitable for adult education, as learners have the opportunity to access and process information at a time and place that suits them [20]. It is defined as a flexible learning approach that enables students to access content and complete assignments at their own pace, without real-time interaction [21].

This study is part of the DeliA project (Delir in Altenpflegeeinrichtungen/Delirium in Nursing Homes) https://osf.io/xkfvh/; funded by the Innovation Committee of the Joint Federal Committee under the funding code 01VSF20003; https://delia.info). The DeliA project aimed to (1) determine the prevalence of delirium in nursing homes in Germany (2) gather data from nurses and general practitioners regarding their knowledge and practices surrounding delirium care (3) develop and evaluate an e-learning program for use in the nursing home setting to address identified gaps.

The e-learning program was therefore designed as an interdisciplinary educational intervention targeting nurses and general practitioners. However, the present manuscript focuses exclusively on the evaluation results for nurses. Therefore, the aim of this study was to test and evaluate an e-learning program to improve delirium-specific knowledge and subjective confidence in the recognition of delirium among nursing home residents by nurses and to assess its suitability. Furthermore, explorative subgroup analyzes were conducted to examine potential differences in outcomes across distinct participant groups.

## Methods

### Development of the e-learning program

The interdisciplinary e-learning program was designed by VM, JS and RP and was technically realised in cooperation with a media agency specialising in science communication in medicine and health. The German-language e-learning program includes the following modules: (1) Definition, complications and consequences; (2) Risk factors and causes; (3) Symptoms and diagnostics; (4) Prevention, therapy and management, and (5) Delirium in the dying phase. Each module contains a 10- to 15-minute video in which one or two experts present the respective content.

The program’s design and structure were developed on the basis of findings from a realist review and an interdisciplinary Delphi study [15, 22]. The Delphi study comprised two rating rounds and an intermediate workshop. Experts were purposively recruited via the DeliA study network and included clinicians with expertise in delirium care (e.g., geriatric nurses, general practitioners, geriatricians, neurologists, intensive care nurses), professionals working in nursing home settings, and academic experts in health and nursing sciences specialising in delirium and/or nursing home care. In both rounds, delirium-specific competencies were rated on a four-point Likert scale, and a priori consensus was defined as ≥ 80% agreement (“very relevant” or “relevant”). Only competencies meeting the predefined consensus criteria were included in the final curriculum [22].

The experts featured in the videos were selected based on their clinical and/or academic expertise in delirium care and long-term care. Most were members of the DeliA consortium, while two experts were recruited from the extended DeliA network. These two experts had also participated as raters in the Delphi study. However, all video content was strictly based on the consensus-derived curriculum.

The videos are supplemented by interactive learning formats such as multiple-choice questions, drag-and-drop elements, and pocket cards. The e-learning program features two filmed case vignettes depicting a hypoactive and a hyperactive subform of delirium in a nursing home resident, as well as a case vignette about delirium in the dying phase. Case vignettes were identified as a key didactic component, as case-based learning enhances understanding of delirium’s clinical manifestations, facilitates the transfer of knowledge into daily practice, and stimulates discussion and reflective exchange among nursing home staff [15]. Additional case vignettes have been included as tests in the e-learning program to train participants in distinguishing between delirium, dementia, and depression. These case vignettes, which were developed in an earlier study of the hospital setting [23], were adapted to the nursing home context during a workshop held as part of the Delphi study [22]. In addition, the e-learning program refers to further literature to enable participants to deepen their knowledge independently. The estimated completion time of the e-learning program ranged from approximately 90 to 120 min. The e-learning program can be used on various devices and runs on the Moodle learning platform (version 4.4.3+). It is accessible via the following link: https://elearning.delia.info/.

### Design and sample

This single-group, non-randomized pre–post pilot study evaluated the e-learning program among nursing home staff from January to March 2025. The intervention was delivered without allocation to treatment arms, randomization, or patient-level clinical interventions. The primary aims of the pilot were to assess feasibility, acceptability, and perceived attractiveness of the program within the nursing home setting, and to generate preliminary insights into potential changes in delirium-specific knowledge and subjective confidence. The convenience sample consisted of nurses working in nursing homes, primarily in the federal states of Bremen, Hamburg, Lower Saxony, and North Rhine-Westphalia. Nursing homes are facilities that provide 24-hour functional support for individuals who require assistance with activities of daily living and instrumental activities of daily living and have identified health and care needs [24]. In Germany, these facilities primarily provide long-term residential care for older adults with complex medical conditions and functional limitations. In addition, some nursing homes may offer short-term transitional, palliative, or hospice care.

Potential participants were recruited primarily through the authors’ networks and contacted by email or telephone. In addition, the e-learning program was advertised at regional network conferences and via newsletters and websites of nonprofit and private organizations. At the start of data collection, interested participants received an informational email, flyers, and posters with a QR code that linked directly to the e-learning program. The first 50 participants to complete the e-learning program received a financial incentive of €100. The incentive was communicated transparently in all recruitment materials as compensation for the time required to complete the e-learning program. Participants were not given any indication that specific study outcomes were expected.

No formal a priori power analysis was conducted. As the study was designed as an exploratory pilot focusing primarily on feasibility, acceptability, and perceived attractiveness no fixed target sample size was predefined. At the planning stage, reliable effect size estimates for this specific setting were not available. Recruitment was therefore conducted over the predefined study period, and the final sample size resulted from participation within this timeframe. Subgroup analyses were considered exploratory. After the e-learning participants agreed to participate in the study, they were asked to provide their sociodemographic and professional information. Specifically, they were also asked whether they held a management function, were involved in direct resident care and had previously completed any delirium training prior to e-learning.

### Questionnaire

Before the nurses could complete the e-learning program, they had to fill out the questionnaire *Knowledge about Delirium (Wissen zum Delir)* [25]. The questionnaire is a German translation and further development of the questionnaire “A questionnaire to determine nurses’ knowledge of delirium and its risk factors” for the hospital setting [26]. The revised German version has previously demonstrated good content validity [25]. The instrument consists of 46 items that can be divided into three sections. The first item of the questionnaire asks for the correct definition of delirium with four possible answers. Subsequently, various screening and assessment instruments must be assigned to *dementia*,* depression*,* delirium* or *none of these*. The majority of the questionnaire comprises delirium-specific statements that must be answered with *“agree”*,* “disagree” or “not sure”*. The overall score can then be based on the number of questions answered correctly.

To verify that the questionnaire was understandable for nurses in nursing homes, cognitive interviews were conducted as a pretest in February 2024 via the Zoom video platform (version 5.14.5; Zoom Video Communications, San Jose, CA, USA). The aim of the cognitive interviews was to examine the comprehensibility of the items and the response processes within the intended target group [27]. A total of five interviews were conducted with nurses from nursing homes who had more than five years of experience in caring for residents. Various cognitive interviewing techniques were applied. With the help of probing techniques, participants were asked to explain technical terms or indicate how confident they felt in answering the question. Using the thinking aloud technique, the interviewees were encouraged to think out loud and, in particular, to verbalise their thoughts when something was unclear [28]. Difficulties in understanding terms or questions were noted, the relevant items were marked, and possible adjustments were suggested directly. All the interviews were recorded and deleted immediately after the data were analyzed.

On the basis of the results of the cognitive interviews, minimal linguistic and semantic adjustments were made to the questionnaire in consultation with VM, JS, and RP. Adjustments were made to the wording (“patient” was replaced with “resident”, and one instrument was exchanged as it was better suited to the nursing home setting). The number of items remained unchanged. Apart from these minor linguistic adaptations, the cognitive interviews indicated that the overall content was comprehensible and contextually appropriate for the nursing home setting.

The adapted version of the questionnaire was named “Knowledge about delirium in nursing homes” and can be found in supplementary material A and B. While the original German revised version had previously demonstrated good content validity, the adaptation for the nursing home context focused on contextual relevance and comprehensibility using structured cognitive interviewing. A full psychometric re-validation of the adapted instrument (e.g., reliability testing or construct validity assessment) was beyond the scope of this exploratory pilot study.

To determine how the participants of the e-learning program would assess their ability to recognize delirium in a resident, they were asked the following question: *How confident do you feel about recognizing delirium?* on a ten-point Likert scale (0 = not confident at all, 5 = moderately confident, 10 = very confident) before and after processing. The posttest was automatically unlocked after completion of all modules. Although the posttest directly followed the intervention within the learning sequence, participants were free to complete it at a time of their choosing. Thus, the interval between completion of the e-learning program and the posttest could vary depending on individual scheduling. After the posttest, the nurses were asked to provide a final evaluation and assessment of the e-learning program and to list the strengths and weaknesses in free text form. The study received approval from the Ethics Committee of the University of Witten/Herdecke (application no. 82/2023).

### Data analysis

The sociodemographic and professional characteristics were presented descriptively using absolute and relative frequencies. To analyze the increase in knowledge and subjective confidence in recognizing delirium, the median and interquartile range (IQR) were calculated for the total sample and relevant subgroups before and after completion of the e-learning program. Responses of “not sure” were coded as incorrect answers for the purpose of calculating the total knowledge score. The Shapiro‒Wilk test was used to check the normal distribution of the dependent samples (α = 0.05). Owing to the deviation from a normal distribution, the nonparametric Wilcoxon signed-rank test was used. P-values were calculated to assess statistical significance. The exact p-values could be determined for the total sample. In some subgroups, asymptotic p-values were calculated because of ties in the ranks. To assess the practical relevance of the observed effects, the effect size was calculated in the form of the rank-biserial correlation coefficient (r), based on the Wilcoxon signed-rank test, with values ranging from − 1 to + 1. The effect sizes were classified as follows: small (*r* ≈ 0.10), medium (*r* ≈ 0.30), or large (*r* ≈ 0.50) [29]. At the item level, the percentage of correct answers in the pre- and posttests and the percentage difference between the two points in time were reported. Given the exploratory pilot design and the large number of individual items (*n* = 46), item-level analyses were interpreted descriptively. Free-text comments evaluating the strengths and weaknesses of the e-learning program were analyzed using a descriptive, inductive categorization approach. The comments were reviewed jointly by VM and JS and grouped into categories based on recurring themes and frequency of occurrence. Categories were defined through discussion and consensus within the research team. Data analysis was performed using Microsoft Excel, R (version 4.4.2; October 31, 2024), RStudio (version 2024.09.1 + 394 “Cranberry Hibiscus”) and a Windows operating system.

## Results

Among the 117 nurses who registered during the survey period, 80 completed both the pre- and posttests, yielding a completion rate of 68.4%. The sample was predominantly female (82.5%), with 80% of participants younger than 50 years. Most had 6–20 years of experience in resident care (57.5%), with the largest subgroups reporting 11–15 years (21.3%) and 6–10 years (20%). Slightly more than half held a management function, and 80% were actively involved in direct resident care. Prior training on delirium was completed by 21.3% of the participants. An overview of participant characteristics is provided in Table 1.

**Table 1:** Sociodemographic and professional characteristics of the participants

Characteristics	Pre and Posttest completed (*n* = 80)
Age	
18–29 years	14 (17.5%)
30–39 years	28 (35%)
40–49 years	22 (27.5%)
50–59 years	13 (16.25%)
60 years and older	3 (3.75%)
Sex	
Female	66 (82.5%)
Male	14 (17.5%)
Specialist nursing training	
Psychogeriatric nursing	2 (2.5%)
Anaesthesia intensive care	2 (2.5%)
Palliative care	4 (5%)
Pain therapy	2 (2.5%)
Job share	
Full-time = 100%	57 (71.25%)
Part-time < 100%	23 (28.75%)
Experience in care of residents	
< 5 years	14 (17.5%)
6–10 years	16 (20%)
11–15 years	17 (21.25%)
16–20 years	12 (15%)
21–25 years	10 (12.5%)
26–30 years	3 (3.75%)
31 years and more	8 (10%)
Management function	
Yes	43 (53.75%)
No	37 (46.25%)
Direct care of residents	
Yes	64 (80%)
No	16 (20%)
Previous delirium training completed*	
Yes	17 (21.25%)
No	63 (78.75%)

Participants could indicate both a management function (e.g., ward manager or nursing service manager) and involvement in direct resident care Therefore, percentages may overlap. Participants without either role were typically employed in administrative, coordinating, or quality management positions

* Refers to any educational activity related to delirium completed before participation in the e-learning program

### Changes in knowledge about delirium

Before completing the e-learning program, the median number of correct answers given by nurses in the pretest was 32 (IQR = 9.25), with a maximum of 46. After completing the e-learning program, the median value increased to 40 (IQR = 7.5) during the posttest. The Wilcoxon signed-rank test was used (V = 222, *p* < 0.001) and yielded a statistically significant result. The calculated effect size of the total sample was *r* = 0.86.

The results from the subgroups show that the median increase in correct answers between the pretest and posttest for the nursing staff in management functions was the highest, Δ = +9, with a p-value = < 0.001 and an effect size of *r* = 0.90. The highest median value in the posttest of 41 was achieved by the subgroup that had already completed other previous delirium training. Here, the reduction in the IQR from 9 to 5 indicates that the values are becoming more homogeneous, with a corresponding p-value of 0.02 and an effect size of *r* = 0.65. Overall, a significant p-value < 0.05 was found in all the subgroups, and the effect sizes were all above *r* = 0.50. This indicates a strong effect size. Subgroups with low median values in the pretest tend to show the greatest increase compared those with higher baseline values. The corresponding data can be found in Table 2.

**Table 2:** Results of changes in delirium-specific knowledge

	*N*	Median (Pretest)	IQR (Pretest)	Median (Posttest)	IQR (Posttest)	*p*-value	*r*
Total	80	32	9.25	40	7.5	< 0.001	0.86
Management function							
Yes	43	31	9	40	9	< 0.001 (~)	0.90
No	37	34	8	40	6	< 0.001 (~)	0.82
Direct care of residents							
Yes	64	32	9	39.5	9	< 0.001	0.84
No	16	35.5	8	40.5	3	< 0.001 (~)	0.94
Previously delirium training completed							
Yes	17	37	9	41	5	0.020 (~)	0.65
No	63	27	8.5	34	8	< 0.001	0.91

*IQR* Interquartile range

(~) The p-value was calculated asymptotically, as it was not possible to calculate the p-value exactly due to the sample size and the presence of ties

### Subjective confidence in recognizing delirium

The median subjective confidence in recognizing delirium among nursing home residents on a 10-point Likert scale in the pretest was 5 (IQR = 2.25). In the posttest, the confidence increased to a median of 7 (IQR = 1). The Wilcoxon signed-rank test was used (V = 84.5, *p* < 0.001), indicating statistical significance. The calculated effect size of the total sample was *r* = 0.92.

When subjective confidence in the ability to recognize delirium was assessed, a significant p-value of < 0.001 and an effect size between 0.90 and 1.00 were achieved for all the subgroups. The subgroup that did not have a management role and was not involved in the direct care of residents showed the greatest median increase, Δ = +3. The two subgroups that did not have a management function and received previous delirium training achieved the highest median score after the posttest (8). The IQR decreased from 4 to 1 among nurses who did not have a management function. The corresponding data can be found in Table 3.

**Table 3:** Results in changes in self-rated confidence in recognizing delirium

	*N*	Median (Pretest)	IQR (Pretest)	Median (Posttest)		IQR (Posttest)	*p*-value	*r*
Total	80	5	2.25		7	1	< 0.001	0.92
Management function								
Yes	43	5	2		7	2	< 0.001 (~)	0.93
No	37	5	4		8	1	< 0.001 (~)	0.93
Direct care of residents								
Yes	64	5	2		7.5	1	< 0.001	0.90
No	16	4	2		7	2.25	< 0.001 (~)	1.00
Previously delirium training completed								
Yes	17	6	4		8	2	< 0.001 (~)	0.91
No	63	5	2		7	1	< 0.001	0.92

(~) The p-value was calculated asymptotically, as it was not possible to calculate the p-value exactly due to the sample size and the presence of ties

### Knowledge about delirium in nursing home items

Table 4 shows the percentage of correct answers for the pre- and posttest at the item level and the corresponding difference. Overall, after the e-learning program was completed by the nursing staff, a percentage increase was achieved for 43 of the 46 items when the results of the pre- and posttests were compared. Three items were answered less correctly after completion (I25, I30, and I34). For five items (I2, I17, I18, I20, and I41), a significant improvement of more than 25% was observed. Three questions were still answered incorrectly in the posttest after the e-learning program was completed (I11, I13 and I14). Details are summarised in Table 4.

**Table 4:** Pre–post changes at item level of the knowledge questionnaire

Item	Item content	Pretest: Correct answers %	Posttest: Correct answers %	Percentage change in correct answers
I1	Definition of delirium	75%	85%	+ 10%
I2	4AT	57.5%	86.25%	+ 28.75%
I3	Back Depressions Inventar	87.5%	91.25%	+ 3.75%
I4	Confusion Assessment Method	56.25%	70%	+ 13.75%
I5	Glasgow Coma Scale	76.25%	78.75%	+ 2.5%
I6	Mini Mental State Examination	82.5%	87.5%	+ 5%
I7	Nursing Delirium Screening Scale	86.25%	87.5%	+ 1.25%
I8	Residents with delirium do not experience perceptual disturbances.	83.75%	85%	+ 1.25%
I9	In residents at risk of delirium, physiological excretion (urination and defecation) can be neglected.	77.5%	85%	+ 7.5%
I10	A resident who is apathetic and difficult to wake does not have delirium.	75%	90%	+ 15%
I11	Residents cannot remember experiencing delirium.	32.5%	38.25%	+ 6.25%
I12	Residents with delirium have a higher mortality rate.	61.25%	78.75%	+ 17.5%
I13	Residents with delirium are more likely to be easily distracted.	35%	47.5%	+ 12.5%
I14	Residents with delirium are physically and/or verbally aggressive.	46.25%	48.75%	+ 2.5%
I15	Residents with an acutely operated femoral neck fracture have a lower risk of developing delirium than residents undergoing a planned hip surgery.	68.75%	80%	+ 11.25%
I16	Residents with moderate dementia, unlike residents with delirium, exhibit attention deficits.	52.5%	61.25%	+ 8.75%
I17	Residents with visual impairments are at an increased risk of delirium.	48.75%	75%	+ 26.25%
I18	Putting on glasses and inserting hearing aids do not have a preventive effect against delirium.	43.75%	70%	+ 26.25%
I19	The risk of delirium increases with age.	81.25%	96.25%	+ 15%
I20	The early removal of intravenous catheters and urinary catheters can help prevent the development of delirium.	37.5%	73.75%	+ 36.25%
I21	Dehydration can be a risk factor for delirium.	88.75%	97.5%	+ 8.75%
I22	Dementia is an important risk factor for delirium.	71.25%	88.75%	+ 17.5%
I23	Involving relatives and close contacts is necessary to prevent delirium	73.75%	88.75%	+ 15%
I24	The treatment of delirium always involves sedation.	81.25%	88.75%	+ 7.5%
I25	Promoting the day-night rhythm is not an essential part of delirium prevention.	76.25%	67.5%	**− 8.75%**
I26	Early mobilization can reduce the risk of delirium.	68.75%	91.25%	+ 22.5%
I27	Infection prevention is part of delirium prevention.	61.25%	83.75%	+ 22.5%
I28	Cognitive activation is part of delirium prevention.	78.75%	96.25%	+ 17.5%
I29	Promoting mobility several times a day is an essential part of delirium prevention.	83.75%	93.75%	+ 10%
I30	Orientation support using calendars, clocks, or photos is not a measure for delirium prevention.	70%	62.5%	**− 7.5%**
I31	Reviewing medication is an important part of delirium prevention and when searching for the cause of a delirium.	93.75%	100%	+ 6.25%
I32	The symptoms of depression can resemble those of hypoactive delirium.	63.75%	81.25%	+ 17.5%
I33	An indwelling urinary catheter reduces the risk of delirium.	75%	87.5%	+ 12.5%
I34	Delirium does not last longer than a few hours.	83.75%	82.5%	**− 1.25%**
I35	Delirium is potentially preventable through nonpharmacological preventive measures.	47.5%	67.5%	+ 20%
I36	Delirium is fundamentally caused by alcohol withdrawal.	85%	85%	+/- 0%
I37	Poor nutritional status increases the risk of delirium.	73.75%	91.25%	+ 17.5%
I38	A disturbed sleep-wake cycle can be a symptom of delirium.	85%	92.5%	+ 7.5%
I39	Adequate nutrition is beneficial in preventing delirium.	83.75%	96.25%	+ 12.5%
I40	A sufficient fluid intake can help prevent delirium.	82.5%	93.75%	+ 11.25%
I41	Hearing impairment increases the risk of delirium.	43.75%	75%	+ 31.25%
I42	It is advisable to restrain confused residents.	87.5%	93.75%	+ 6.25%
I43	The more medications residents take, the higher the risk of delirium.	75%	95%	+ 20%
I44	Pain has no impact on the development of delirium.	77.5%	91.25%	+ 13.75%
I45	Fluctuations between being oriented and disoriented are not typical for delirium.	43.75%	63.75%	+ 20%
I46	Behavioral changes throughout the day are typical for delirium.	70%	82.5%	+ 12.5%

^1^The questionnaire was translated from German into English by the author without claiming to provide a validated instrument translation. The translation was conducted solely for the purpose of presentation in this publication and requires further validation before practical application

### Evaluation

The analysis of the free-text comments resulted in five main categories reflecting participants’ perceptions of the e-learning program: *practical relevance and applicability*, *didactic design and structure*, *flexibility and accessibility*, *target group suitability*, and *technical or structural challenges*.

### Practical relevance and applicability

Participants described the e-learning program as highly practical and applicable to everyday nursing home practice. The realistic case vignettes and pocket cards were particularly appreciated, as they supported the transfer of theoretical knowledge into daily care routines. Several respondents emphasized that the program represents a valuable alternative to traditional classroom-based training and is well suited as a structured educational intervention.

### Didactic design and structure

The didactic implementation was frequently praised. Participants highlighted the clear structure, small learning units, interactive elements, and the possibility of repeating content. The combination of theoretical input and practical case examples was perceived as especially beneficial. Many respondents described the program as informative, comprehensible, and well designed.

### Flexibility and accessibility

Flexibility emerged as one of the most frequently mentioned strengths. The possibility to complete modules at an individual pace, integrate learning into shift work (e.g., night shifts), and access content repeatedly from home or the workplace was considered highly advantageous.

### Target group suitability

Participants noted that the e-learning format could potentially reach a broad range of nursing staff, including nursing assistants and volunteers. However, opinions differed regarding its suitability for all target groups. Some respondents suggested that older staff members might require additional technical support and that the scope of content could be challenging for participants without prior knowledge of delirium.

### Technical and structural challenges

Despite the generally positive evaluation, technical barriers were reported. Some participants felt overwhelmed by the learning platform and expressed a need for a more user-friendly introduction. The requirement for self-motivation and independent time management was also identified as a potential challenge.

## Discussion

In this study, an e-learning program on delirium in nursing homes was piloted and evaluated for nurses. After the e-learning program was completed, increases in delirium-specific knowledge, greater subjective self-confidence in recognizing delirium among residents, and positive and negative results of the program evaluation were recorded. To the best of our knowledge, this is the first empirical study in the German context to address the topic of knowledge promotion of delirium in nursing homes.

The results of this pilot study suggest that the e-learning program may contribute to improved delirium-specific knowledge among nursing staff in nursing homes. The item-level analysis of the “Knowledge about Delirium in nursing homes” questionnaire indicated that the e-learning program was associated with increased awareness of delirium-specific care, as numerous items related to risk factors, prevention, and detection showed improvement. In particular, awareness appeared to increase regarding key aspects such as delirium-associated mortality, preventive strategies such as the early removal of catheters, and the use of screening tools, areas that align with current recommendations emphasising the pivotal role of nurses in delirium prevention and management [30].

The large effect sizes observed for both knowledge and subjective confidence indicate not only statistical significance but also potential educational relevance. In practical terms, participants showed substantial gains in delirium-related knowledge and perceived recognition confidence. In the nursing home context, where delirium is frequently underrecognized, even moderate improvements in awareness and confidence may contribute to earlier identification. However, improved knowledge and increased self-reported confidence do not automatically translate into changes in clinical practice. Previous educational studies have demonstrated that gains in test performance do not necessarily result in more accurate delirium recognition or consistent implementation in routine care [31]. Therefore, the extent to which the observed improvements influence actual care processes and resident outcomes remains to be examined in future research.

The qualitative evaluation findings provide additional context for these quantitative improvements. Frequently praised elements such as realistic case vignettes, interactive components, and the possibility to repeat content may have facilitated deeper engagement with the topic and supported the transfer of theoretical knowledge into practice. Case-based learning, in particular, may enhance clinical reasoning in complex situations such as delirium recognition [15].

Conversely, reported technical barriers and feelings of being overwhelmed by the learning platform may have limited engagement for some participants [32]. It is conceivable that such challenges moderated learning effects in certain subgroups. Future implementations should therefore aim to further optimise usability and provide additional technical support to maximise educational impact across diverse staff groups.

Interestingly, three items showed a decrease in correct responses following the intervention. While these items were negatively worded and may have posed formal challenges, the finding also suggests that certain content areas may require clearer emphasis or more explicit clarification within the e-learning modules. Future revisions should therefore review these aspects to reduce potential misunderstandings.

In hospital settings, e-learning programs aimed at increasing awareness and enhancing knowledge about delirium have been implemented across various departments [33, 34]. A persistent knowledge deficit among health care staff regarding delirium remains a significant barrier to the development of structured and effective delirium management strategies [35]. This challenge is equally relevant to the nursing home context. It can be assumed that many episodes of delirium among highly vulnerable nursing home residents remain undetected because health care professionals have limited delirium-specific expertise and insufficient diagnostic skills [36]. Additionally, there is a widespread tendency to misinterpret acute confusion in older adults as a normal and inevitable aspect of the ageing process or the progression of dementia, which further contributes to the underrecognition of delirium [37].

The e-learning program developed in the DeliA project focused specifically on delirium in nursing homes and was therefore tailored to this setting [15, 22]. The primary goal in designing the e-learning program was to focus on the target group to increase the acceptance rate [38]. This also explains why no existing e-learning programs from other areas were used, as the terminology and language needed to be adapted to the nursing home context, which is particularly important for target-group-specific learning.

The pilot e-learning program represents a first step towards further training on delirium in German nursing homes. It is undisputed that e-learning alone is not sufficient to bring about a change in behavior in the direct care of residents and to enhance clinical skills in delirium detection. A particularly valuable improvement is the combination of e-learning programs with face-to-face training, commonly referred to as a blended learning format [39]. Notably, the nursing staff in the pilot study who had previously completed delirium training benefited most from e-learning. It is assumed that a portion of this sample had already participated in the prevalence study conducted as part of the DeliA project. In preparation for this prevalence study, the nursing staff received a one-day face-to-face training delivered by a nurse, a general practitioner, and a pharmacist. The training consisted of structured lectures on delirium pathogenesis, symptoms, risk factors, and pharmacotherapy [14]. In line with the Predisposing, Reinforcing, and Enabling Constructs in Educational Diagnosis and Evaluation (PRECEDE) model [40], multimodal educational programs for delirium prevention could be developed. On the basis of the PRECEDE model, educational programs should not only provide knowledge (predisposing factors) but also promote practical application and interaction (enabling factors) and be repeated regularly (reinforcing factors). The PRECEDE model could therefore serve as an explanatory framework for why the subgroup that completed both in-person training and e-learning benefitted from the active face-to-face component and repetition. Such structured and repetitive training formats delivered by interprofessional teams could be promising in nursing homes for sustainably anchoring the topic of delirium [41]. The results of the e-learning evaluation also indicate that a stronger focus on interaction and collegial exchange would be useful, not least to better meet the needs of different learning types.

Nursing staff in management and leadership functions also participated in the e-learning program and were able to gain delirium-specific knowledge. The high participation rate of nurses with management functions shows that there is great interest in this topic. Raising awareness at the management level is particularly important for establishing a long-term reduction in delirium and thus improving the quality of care for residents [15, 35]. Additionally, this provides nursing managers with insight into a cost-, time- and resource-efficient e-learning program, which provides relevant information for later decisions on implementation. The evaluation of the e-learning program also confirms that, in addition to nursing professionals, nursing assistants and other unskilled support staff must also receive training on delirium [42]. Specifically, certified nursing assistants, who have intensive contact with residents and act as a link to nursing staff, have a specific need for knowledge about delirium [43], which must be addressed through appropriate learning formats [44]. In the evaluation, nurses were divided in their opinions on whether DeliA e-learning was also suitable for nursing assistants, particularly with regard to the severity level.

Addressing the phenomenon of delirium in nursing homes is highly relevant in Germany, as nursing home staff report a specific need for knowledge on the subject, and nursing home residents are very susceptible to developing delirium. This is exacerbated by demographic developments, as delirium episodes become more frequent as the population ages [45]. Furthermore, the topic of delirium in nursing homes has received little to no attention in research, society, or politics to date and must be given much greater focus at all levels.

### Limitations

This pilot study is among the first in Germany to address the phenomenon of delirium in nursing homes and takes a first step towards raising awareness with a target group-specific e-learning program. As an exploratory pilot study, the sample size of 80 nursing professionals provided initial insights to inform further refinement of the e-learning program. The sample is characterised by self-selection of participants i.e., nursing professionals who were particularly interested in the topic were more likely to complete the e-learning program. Financial incentives must also be considered, as they influenced participation in the study and likely led to nurses completing the e-learning program who otherwise might not have done so. This may have led to an overestimation of the observed effects, as participants with higher intrinsic motivation or prior interest in delirium may have been more engaged with the learning material and more receptive to knowledge acquisition. Consequently, the results may not fully reflect the impact of the intervention in a less motivated or more heterogeneous population. Furthermore, the interpretation of data at the subgroup level must be performed with caution, as the sample size was very small. The small subgroup sizes reduce statistical precision and increase the likelihood of unstable effect estimates. As no formal a priori power calculation or predefined target sample size was established, the final sample depended on participation within the recruitment period, and the study was not statistically powered to detect small effects or subgroup differences with high precision. Therefore, subgroup findings should be interpreted as exploratory rather than confirmatory and cannot be generalised to specific professional groups without further validation in adequately powered studies. Although significant improvements with large effect sizes were observed in the overall sample, the robustness of subgroup analyses remains limited. Future controlled studies should perform formal sample size calculations based on the effect sizes observed in the present study. In addition, the nursing homes differed considerably in terms of size and clientele. With respect to recruitment, it must be taken into account that the email addresses did not directly reach the nursing professionals instead, the messages always had to be forwarded to the interested parties through the management of the nursing home, which must be considered a barrier.

Another limitation is that pre- and posttests almost always lead to improvements in the intervention group, especially when the measurement is taken immediately after the intervention. Given that this is a pilot study, future studies on the impact of delirium training should include more time points for data collection. Moreover, the immediate post-intervention assessment does not allow conclusions about the sustainability of knowledge and confidence gains. It remains unclear whether the observed improvements persist over time or translate into sustained behavioural changes in clinical practice. Long-term follow-up assessments are therefore essential to evaluate retention effects and practical implementation.

Although the “Knowledge about delirium in nursing homes” questionnaire showed good content validity, some items contained negative wording and formal stumbling blocks. It is striking that all items that were rated worse in the comparison between the pre- and posttests were negatively worded. The incorrect answers may partly be due to misunderstandings related to the negatively worded items. However, it cannot be ruled out that certain content areas require clearer emphasis within the e-learning modules.

## Conclusions

Residents of nursing homes are at increased risk of developing delirium, while nursing staff report a clear need for delirium-specific expertise. The e-learning program evaluated in this pilot study represents a promising foundation for structured training and awareness-raising on delirium in nursing homes. However, given the exploratory design and methodological limitations, the findings should be interpreted as preliminary.

Following further refinement, the program could be embedded in a multimodal training approach that supports the sustainable integration of delirium management into routine nursing home practice. Future research should include larger and more diverse samples, formal sample size calculations, and long-term follow-up assessments to further validate effectiveness and implementation potential.

The integration of tailored e-learning into nursing education and continuing professional development may contribute to earlier awareness and improved recognition of delirium in nursing home residents. Strengthening delirium-specific knowledge is a central prerequisite for the development and implementation of structured delirium management processes and may support the improvement of professional delirium care in this setting.

## Supplementary Information

Supplementary Material 1.

Supplementary Material 2.

## Data Availability

The datasets used and/or analyzed during the current study are available from the corresponding author on reasonable request.

## Abbreviations

DeliA: Delir in Altenpflegeeinrichtungen
DSM-V-TR: Diagnostic and Statistical Manual of Mental Disorders, Fifth Edition, Text-Revision
IQR: Interquartile range
PRECEDE: Predisposing, Reinforcing, and Enabling Constructs in Educational Diagnosis and Evaluation
