# Supplementary material for: Improving delirium knowledge and recognition confidence in nursing homes through an e-learning program: a pre–post study
Source: BMC Med Educ. 2026 Apr 25;26:680. doi: 10.1186/s12909-026-09297-2 (PMC13113018; doi:10.1186/s12909-026-09297-2)
Supplement: Supplementary file 1 — Supplementary Material 1. [file 12909_2026_9297_MOESM1_ESM.docx]

**Wissen zum Delir in Altenpflegeeinrichtungen**

**Welche der folgenden Symptomkomplexe beschreibt oder definiert das Delir am besten? (Wählen Sie die zutreffendste Antwort)**

a) O Vergesslich/ Amnestisch, schläfrig, plötzlich einsetzende Inkontinenz, unkontrollierter Speichelfluss, unorganisiertes Denken

**b) O** Akute Verwirrheit, flukturierender (schwankender) Aufmerksamkeits- und Bewusstseinszustand, unorganisiertes Denken, veränderter Bewusstseinsgrad

c) O Angstzustände, Diaphorese (Schwitzen), Zittern, Muskelschwäche, Dysphasie (Schluckstörung), veränderter Erregungszustand

d) O Langsames Einsetzen von Verwirrung, Gedächtnisverlust, Desorientierung, mangelnder Spontanität, Persönlichkeitsveränderung

**Die folgenden Bewertungsinstrumente werden häufig verwendet, um bestimmte Erkrankungen zu erkennen. Ordnen Sie die jeweiligen Bewertungsinstrumente der/den passendsten Erkrankung/en zu.** Beachten Sie, dass „Keine davon“ möglicherweise die richtige Antwort ist. Sie können für jedes Bewertungsinstrument mehr als eine Erkrankung auswählen.

| **Bewertungsinstrumente** | **Delir** | **Demenz** | **Depression** |  | **Keine**  **davon** |
| --- | --- | --- | --- | --- | --- |
| **Mini Mental State Examination (MMSE)** (Test zur Erfassung der kognitiven Fähigkeiten) | O | **O** | O |  | O |
| **4AT** | **O** | O | O |  | O |
| **Nursing Delirium Screening Scale (Nu-DESC)** | **O** | O | O |  | O |
| **Glasgow Coma Scale (GCS)** | O | O | O |  | **O** |
| **Confusion Assessment Method (CAM)** | **O** | O | O |  | O |
| **Beck-Depressions-Inventar** | O | O | **O** |  | O |

**Bitte beantworten Sie die nachfolgenden Aussagen durch Zustimmung, Ablehnung oder unsicher**

| **Schwankungen zwischen orientiert und desorientiert sind nicht typisch für ein Delir.** | Stimme zu | Stimme nicht zu |  | Unsicher |
| --- | --- | --- | --- | --- |
| **Die Symptome einer Depression können einem hypoaktiven Delir ähneln.** | Stimme zu | Stimme nicht zu |  | Unsicher |
| **Die Behandlung eines Delirs beinhaltet immer die Sedierung.** | Stimme zu | Stimme nicht zu |  | Unsicher |
| **Bewohner:innen können sich nicht an ein Delir erinnern.** | Stimme zu | Stimme nicht zu |  | Unsicher |
| **Bewohner:innen mit einer mittelgradigen Demenz fallen im Gegensatz zu Patienten mit einem Delir durch eine Aufmerksamkeitsstörung auf.** | Stimme zu | Stimme nicht zu |  | Unsicher |
| **Bewohner:innen mit einer akut operierten Schenkelhalsfraktur haben ein niedrigeres Risiko ein Delir zu entwickeln als Bewohner:innen mit einer planmäßigen Hüftoperation.** | Stimme zu | Stimme nicht zu |  | Unsicher |
| **Ein Delir dauert nicht länger als ein paar Stunden.** | Stimme zu | Stimme nicht zu |  | Unsicher |
| **Das Delirrisiko steigt mit zunehmendem Alter.** | Stimme zu | Stimme nicht zu |  | Unsicher |
| **Bewohner:innen mit Seheinschränkung sind einem erhöhten Delirrisiko ausgesetzt.** | Stimme zu | Stimme nicht zu |  | Unsicher |
| **Je mehr Medikamente Bewohner:innen, einnehmen, desto höher ist das Delirrisiko.** | Stimme zu | Stimme nicht zu |  | Unsicher |
| **Ein Blasenverweilkatheter reduziert das Delirrisiko.** | Stimme zu | Stimme nicht zu |  | Unsicher |
| **Ein schlechter Ernährungszustand erhöht das Delirrisiko.** | Stimme zu | Stimme nicht zu |  | Unsicher |
| **Demenz ist ein wichtiger Risikofaktor für ein Delir.** | Stimme zu | Stimme nicht zu |  | Unsicher |
| **Dehydratation kann ein Risikofaktor für ein Delir sein.** | Stimme zu | Stimme nicht zu |  | Unsicher |
| **Eine Einschränkung des Hörens erhöht das Risiko für ein Delir.** | Stimme zu | Stimme nicht zu |  | Unsicher |
| **Bewohner:innen die teilnahmslos und schwer zu erwecken sind, haben kein Delir.** | Stimme zu | Stimme nicht zu |  | Unsicher |
| **Bewohner:innen mit einem Delir sind körperlich und/oder verbal aggressiv.** | Stimme zu | Stimme nicht zu |  | Unsicher |
| **Ein Delir wird grundsätzlich durch einen Alkoholentzug verursacht.** | Stimme zu | Stimme nicht zu |  | Unsicher |
| **Bewohner:innen mit einem Delir haben eine höhere Sterblichkeit.** | Stimme zu | Stimme nicht zu |  | Unsicher |
| **Verhaltensveränderungen im Tagesverlauf sind typisch für ein Delir.** | Stimme zu | Stimme nicht zu |  | Unsicher |
| **Bewohner:innen mit einem Delir können mit einer höheren Wahrscheinlichkeit leicht abgelenkt werden.** | Stimme zu | Stimme nicht zu |  | Unsicher |
| **Bei Bewohner:innen mit Delir treten keine Wahrnehmungsstörungen auf.** | Stimme zu | Stimme nicht zu |  | Unsicher |
| **Ein veränderter Schlaf- / Wachrhythmus kann ein Symptom für ein Delir sein.** | Stimme zu | Stimme nicht zu |  | Unsicher |

| **Das Aufsetzen der Brille und das Einsetzen der Hörgeräte wirken nicht delirpräventiv.** | Stimme zu | Stimme nicht zu |  | Unsicher |
| --- | --- | --- | --- | --- |
| **Ein Delir ist durch nicht-pharmakologische Präventionsmaßnahmen potentiell vermeidbar.** | Stimme zu | Stimme nicht zu |  | Unsicher |
| **Die kognitive Aktivierung ist Teil der Delirprävention.** | Stimme zu | Stimme nicht zu |  | Unsicher |
| **Eine ausreichende Flüssigkeitsaufnahme kann einem Delir entgegenwirken.** | Stimme zu | Stimme nicht zu |  | Unsicher |
| **Es ist empfehlenswert, verwirrte Bewohner:innen zu fixieren.** | Stimme zu | Stimme nicht zu |  | Unsicher |
| **Die Infektionsprävention ist Teil der Delirprävention.** | Stimme zu | Stimme nicht zu |  | Unsicher |
| **Das frühzeitige Entfernen von Venenverweilkatheter und Blasenkatheter können der Entstehung eines Delirs entgegenwirken.** | Stimme zu | Stimme nicht zu |  | Unsicher |
| **Die mehrmals tägliche Förderung der Mobilität ist eine wesentlicher Teil der Delirprävention.** | Stimme zu | Stimme nicht zu |  | Unsicher |
| **Die Orientierungsförderung durch Kalender/ Uhr oder Fotos ist keine Maßnahme der Delirprävention.** | Stimme zu | Stimme nicht zu |  | Unsicher |
| **Schmerzen haben keinen Einfluss auf die Delirentstehung.** | Stimme zu | Stimme nicht zu |  | Unsicher |
| **Die Prüfung der Medikation ist ein wichtiger Bestandteil der Delirpävention und bei der Suche nach der Ursache eines Delirs.** | Stimme zu | Stimme nicht zu |  | Unsicher |
| **Die Frühmobilisation kann das Delirrisiko verringern.** | Stimme zu | Stimme nicht zu |  | Unsicher |
| **Der Einbezug von An- und Zugehörigen ist notwendig, um ein Delir zu vermeiden.** | Stimme zu | Stimme nicht zu |  | Unsicher |
| **Eine ausreichende Ernährung ist von Vorteil bei der Vermeidung des Delirs.** | Stimme zu | Stimme nicht zu |  | Unsicher |
| **Bei Delir gefährdeten Bewohner:innen ist die physiologische Ausscheidung (Miktion und Defäkation) zu vernachlässigen.** | Stimme zu | Stimme nicht zu |  | Unsicher |
| **Die Förderung des Tag-Nacht-Rhythmus ist kein wesentlicher Bestandteil der Delirprävention.** | Stimme zu | Stimme nicht zu |  | Unsicher |

**This questionnaire is an adapted version of the instrument *Wissen zum Delir* originally published in: Zilezinski M, Lohrmann R, Hauß A, Bergjan M. Development and content validity of a questionnaire to assess knowledge about delirium. Z Gerontol Geriatr. 2023;56(2):132–138. doi:10.1007/s00391-022-02015-9.**
