# Supplementary material for: Improving delirium knowledge and recognition confidence in nursing homes through an e-learning program: a pre–post study
Source: BMC Med Educ. 2026 Apr 25;26:680. doi: 10.1186/s12909-026-09297-2 (PMC13113018; doi:10.1186/s12909-026-09297-2)
Supplement: Supplementary file 2 — Supplementary Material 2. [file 12909_2026_9297_MOESM2_ESM.docx]

**Knowledge of Delirium in Nursing Homes**

Which of the following symptom complexes best describes or defines delirium? *(Select the most appropriate answer)*

a) ⭘ Forgetfulness/amnesia, drowsiness, sudden onset of incontinence, excessive salivation, disorganized thinking
b) ⭘ Acute confusion, fluctuating level of attention and consciousness, disorganized thinking, altered level of consciousness
c) ⭘ Anxiety, diaphoresis (sweating), tremor, muscle weakness, dysphagia (difficulty swallowing), altered arousal
d) ⭘ Gradual onset of confusion, memory loss, disorientation, reduced spontaneity, personality changes

The following assessment instruments are commonly used to identify specific conditions. Please assign each instrument to the most appropriate condition(s). Note that “None of the above” may be the correct answer. You may select more than one condition for each instrument.

| **Assessment Instruments** | **Delirium** | **Dementia** | **Depression** |  | **None of the above** |
| --- | --- | --- | --- | --- | --- |
| **Mini Mental State Examination (MMSE) *(assessment of cognitive function)*** | O | **O** | O |  | O |
| **4AT** | **O** | O | O |  | O |
| **Nursing Delirium Screening Scale (Nu-DESC)** | **O** | O | O |  | O |
| **Glasgow Coma Scale (GCS)** | O | O | O |  | **O** |
| **Confusion Assessment Method (CAM)** | **O** | O | O |  | O |
| **Beck Depression Inventory (BDI)** | O | O | **O** |  | O |

Please respond to the following statements by indicating agreement, disagreement, or uncertainty.

| **Residents with delirium do not experience perceptual disturbances.** | Agree | Disagree | Uncertain |
| --- | --- | --- | --- |
| **In residents at risk of delirium, physiological excretion (urination and defecation) can be neglected.** | Agree | Disagree | Uncertain |
| **A resident who is apathetic and difficult to wake does not have delirium.** | Agree | Disagree | Uncertain |
| **Residents cannot remember experiencing delirium.** | Agree | Disagree | Uncertain |
| **Residents with delirium have a higher mortality rate.** | Agree | Disagree | Uncertain |
| **Residents with delirium are more likely to be easily distracted.** | Agree | Disagree | Uncertain |
| **Residents with delirium are physically and/or verbally aggressive.** | Agree | Disagree | Uncertain |
| **Residents with an acutely operated femoral neck fracture have a lower risk of developing delirium than residents undergoing a planned hip surgery.** | Agree | Disagree | Uncertain |
| **Residents with moderate dementia, unlike residents with delirium, exhibit attention deficits.** | Agree | Disagree | Uncertain |
| **Residents with visual impairments are at an increased risk of delirium.** | Agree | Disagree | Uncertain |
| **Putting on glasses and inserting hearing aids do not have a preventive effect against delirium.** | Agree | Disagree | Uncertain |
| **The risk of delirium increases with age.** | Agree | Disagree | Uncertain |
| **The early removal of intravenous catheters and urinary catheters can help prevent the development of delirium.** | Agree | Disagree | Uncertain |
| **Dehydration can be a risk factor for delirium.** | Agree | Disagree | Uncertain |
| **Dementia is an important risk factor for delirium.** | Agree | Disagree | Uncertain |
| **Involving relatives and close contacts is necessary to prevent delirium** | Agree | Disagree | Uncertain |
| **The treatment of delirium always involves sedation.** | Agree | Disagree | Uncertain |
| **Promoting the day-night rhythm is not an essential part of delirium prevention.** | Agree | Disagree | Uncertain |
| **Early mobilization can reduce the risk of delirium.** | Agree | Disagree | Uncertain |
| **Infection prevention is part of delirium prevention.** | Agree | Disagree | Uncertain |
| **Cognitive activation is part of delirium prevention.** | Agree | Disagree | Uncertain |
| **Promoting mobility several times a day is an essential part of delirium prevention.** | Agree | Disagree | Uncertain |
| **Orientation support using calendars, clocks, or photos is not a measure for delirium prevention.** | Agree | Disagree | Uncertain |
| **Reviewing medication is an important part of delirium prevention and when searching for the cause of a delirium.** | Agree | Disagree | Uncertain |
| **The symptoms of depression can resemble those of hypoactive delirium.** | Agree | Disagree | Uncertain |
| **An indwelling urinary catheter reduces the risk of delirium.** | Agree | Disagree | Uncertain |
| **Delirium does not last longer than a few hours.** | Agree | Disagree | Uncertain |
| **Delirium is potentially preventable through nonpharmacological preventive measures.** | Agree | Disagree | Uncertain |
| **Delirium is fundamentally caused by alcohol withdrawal.** | Agree | Disagree | Uncertain |
| **Poor nutritional status increases the risk of delirium.** | Agree | Disagree | Uncertain |
| **A disturbed sleep-wake cycle can be a symptom of delirium.** | Agree | Disagree | Uncertain |
| **Adequate nutrition is beneficial in preventing delirium.** | Agree | Disagree | Uncertain |
| **A sufficient fluid intake can help prevent delirium.** | Agree | Disagree | Uncertain |
| **Hearing impairment increases the risk of delirium.** | Agree | Disagree | Uncertain |
| **It is advisable to restrain confused residents.** | Agree | Disagree | Uncertain |
| **The more medications residents take, the higher the risk of delirium.** | Agree | Disagree | Uncertain |
| **Pain has no impact on the development of delirium.** | Agree | Disagree | Uncertain |
| **Fluctuations between being oriented and disoriented are not typical for delirium.** | Agree | Disagree | Uncertain |
| **Behavioral changes throughout the day are typical for delirium.** | Agree | Disagree | Uncertain |

**This questionnaire is an adapted version of the instrument *Wissen zum Delir* originally published in: Zilezinski M, Lohrmann R, Hauß A, Bergjan M. Development and content validity of a questionnaire to assess knowledge about delirium. Z Gerontol Geriatr. 2023;56(2):132–138. doi:10.1007/s00391-022-02015-9.**
